# Decreased Serum and Tissue Levels of Isthmin‐1 in Patients With Idiopathic Granulomatous Mastitis: A Case–Control Study

**DOI:** 10.1155/jimr/6368073

**Published:** 2025-11-20

**Authors:** Ahmet Karatas, Burak Oz, Ramazan Fazıl Akkoc, Ibrahim Hanifi Ozercan, Suleyman Serdar Koca

**Affiliations:** ^1^ Department of Rheumatology, Faculty of Medicine, Firat University, Elazig, Türkiye, firat.edu.tr; ^2^ Department of Anatomy, Faculty of Medicine, Firat University, Elazig, Türkiye, firat.edu.tr; ^3^ Department of Pathology, Faculty of Medicine, Firat University, Elazig, Türkiye, firat.edu.tr

**Keywords:** biomarker, diagnosis, idiopathic granulomatous mastitis, inflammation, Isthmin-1

## Abstract

**Background:**

Idiopathic granulomatous mastitis (IGM) is a chronic inflammatory breast disorder with unclear etiology. Isthmin‐1 (ISM1), a secreted protein with anti‐inflammatory properties, has not been previously studied in IGM.

**Objective:**

This study aimed to compare serum and tissue ISM1 levels between IGM patients and healthy controls, and to assess its diagnostic potential.

**Methods:**

This case–control study included 30 women with histopathologically confirmed IGM and 30 age‐matched controls undergoing breast reduction surgery. Serum and tissue ISM1 levels were measured using ELISA. Receiver operating characteristic (ROC) analysis assessed the diagnostic performance of serum ISM1.

**Results:**

ISM1 concentrations were significantly lower in IGM patients compared to controls in both serum (541.42 ± 191.01 vs. 1139.19 ± 698.43 pg/mL; *p* = 0.019) and tissue (511.07 ± 188.16 vs. 778.24 ± 261.98 pg/mL; *p*  < 0.001). ROC analysis demonstrated moderate diagnostic accuracy (area under the curve [AUC]: 0.768, 95% CI: 0.651–0.885; optimal cutoff: 676.13 pg/mL; sensitivity: 66.7%; specificity: 83.7%). Standard inflammatory markers showed no significant differences between groups.

**Conclusions:**

Reduced ISM1 levels in IGM patients suggest potential involvement in disease pathogenesis. While serum ISM1 shows promise as a supportive biomarker, larger studies, including other inflammatory breast conditions, are needed to confirm specificity and clinical utility.

## 1. Introduction

Idiopathic granulomatous mastitis (IGM) is a rare, chronic inflammatory breast disease predominantly affecting women of reproductive age [1, 2]. Histologically characterized by noncaseating granulomas composed of epithelioid histiocytes, multinucleated giant cells, and lymphocytes around breast lobules, IGM presents a diagnostic and therapeutic challenge [3, 4]. Despite extensive research, the etiology remains unclear, with proposed mechanisms, including autoimmune processes, hormonal factors, latent infections, and genetic susceptibility [3, 4].

Clinical management of IGM remains challenging, with corticosteroids and immunosuppressants as first‐line therapies and surgery reserved for selected cases [5–7]. The disease typically follows a chronic, relapsing course with recurrence rates of 16%–50% [3, 5]. This unpredictable nature and significant impact on quality of life underscore the need for biomarkers to guide diagnosis and treatment.

Isthmin‐1 (ISM1), initially identified in brain development [8], has emerged as a regulator of immune and inflammatory processes. Through interactions with surface receptors, including αvβ5 integrin and glucose‐regulated protein 78 (GRP78), ISM1 influences angiogenesis, apoptosis, and immune signaling [9–11]. Recent evidence suggests ISM1 regulates inflammation and immune responses across multiple organs [10, 12, 13], though its role in breast tissue remains undefined.

Given the immune dysregulation in IGM and ISM1’s anti‐inflammatory properties, we hypothesized that ISM1 levels might be altered in IGM patients. This study aimed to compare serum and tissue ISM1 levels between IGM patients and healthy controls and evaluate the diagnostic potential of serum ISM1.

## 2. Materials and Methods

### 2.1. Study Design and Ethical Approval

This case–control study was approved by the Firat University Ethics Committee (Approval Number: 26962/24) and conducted according to the Declaration of Helsinki. All participants provided written informed consent.

### 2.2. Study Population

The study included 30 women with histopathologically confirmed IGM and 30 age‐matched women undergoing elective breast reduction surgery. IGM diagnosis required typical histological findings (noncaseating granulomas, epithelioid histiocytes, multinucleated giant cells, and lymphocytes) after excluding infections and other granulomatous diseases.

*Inclusion criteria:* (1) age >18 years; (2) clinical and radiological findings consistent with IGM; (3) histological confirmation of IGM.

*Exclusion criteria:* (1) follow‐up <6 months; (2) infectious causes (tuberculosis and fungal infections); (3) systemic granulomatous diseases (sarcoidosis and vasculitis); (4) malignancy.

Sample size calculation using G^∗^Power 3.1.9.7 (effect size: 0.7, *α* = 0.05, power = 0.80) indicated 26 participants per group; 30 were enrolled to account for potential attrition.

### 2.3. Clinical and Laboratory Assessment

Demographic data (age, BMI, obstetric history, breastfeeding status) and clinical features (symptom duration, pain, erythema, mass, fistula, abscess) were collected from medical records. Laboratory parameters included erythrocyte sedimentation rate (ESR), C‐reactive protein (CRP), and complete blood count.

### 2.4. Sample Collection and Processing

#### 2.4.1. Tissue Samples

IGM specimens were obtained from tissue biopsies performed for diagnosis. Control tissues were from breast reduction surgeries. Samples were fixed in 10% formalin, paraffin‐embedded, and archived. Representative blocks were selected after pathological review.

For analysis, tissues were homogenized in phosphate buffer (pH 7.4, 1:10 w/v) as previously described [14], centrifuged (10,000 rpm, 5 min, 4°C), and supernatants stored at −20°C. Protein concentration was determined using the Bradford method.

#### 2.4.2. Serum Samples

Venous blood samples were collected from fasting participants. Serum was separated by centrifugation and stored at −20°C until analysis.

### 2.5. ISM1 Measurement

ISM1 levels were quantified in serum and tissue homogenates using a commercial Human ISM1 ELISA Kit (Sunred Biological Technology Co., Shanghai, China) according to the manufacturer’s instructions. The kit specifications were detection range 10–3000 ng/L, sensitivity 9.625 ng/L, intra‐assay CV <10%, and interassay CV <12%. All samples were analyzed in duplicate, and mean values were used for statistical analysis.

### 2.6. Statistical Analysis

Statistical analyses were performed using IBM SPSS Statistics version 26.0 (IBM Corp., Armonk, NY, USA). Normality was assessed using Shapiro–Wilk test and Q–Q plots. Data are presented as mean ± standard deviation (SD) for normally distributed variables and median (interquartile range) for nonparametric variables.

Between‐group comparisons were performed using Student’s *t*‐test or Mann–Whitney *U* test for continuous variables and chi‐square or Fisher’s exact test for categorical variables. Diagnostic performance of serum ISM1 was evaluated using receiver operating characteristic (ROC) analysis, calculating area under the curve (AUC), optimal cutoff value (Youden index), sensitivity, and specificity. *p*‐Values <0.05 were considered statistically significant.

## 3. Results

### 3.1. Patient Characteristics

Clinical characteristics of IGM patients are presented in Table 1. Mean symptom duration was 16.6 ± 27.5 months. The most common presentation was painful breast mass (86.7%), followed by erythema (73.3%) and axillary lymphadenopathy (30%). Nipple retraction occurred in 16.7% and fistula formation in 26.7%. Disease distribution was left‐sided in 59%, right‐sided in 31%, and bilateral in 10%. Overall, 90% of patients had breastfed within the past 5 years.

**Table 1:** Clinical characteristics of IGM patients (*n* = 30).

Characteristic	Value
Symptom duration (months)	16.6 ± 27.5
Breastfeeding duration (months)	14.2 ± 6.8
Number of births	1–8/3 (min–max/median)
Recent breastfeeding (<5 years), *n* (%)	27 (90.0)
Painful mass, *n* (%)	26 (86.7)
Erythema, *n* (%)	22 (73.3)
Axillary lymphadenopathy, *n* (%)	9 (30.0)
Nipple retraction, *n* (%)	5 (16.7)
Fistula formation, *n* (%)	8 (26.7)
Disease distribution:	
‐ Left‐sided, *n* (%)	17 (59.0)
‐ Right‐sided, *n* (%)	9 (31.0)
‐ Bilateral, *n* (%)	3 (10.0)

Abbreviation: IGM, idiopathic granulomatous mastitis.

### 3.2. Demographic and Laboratory Parameters

Table 2 compares demographic and laboratory parameters between groups. No significant differences were observed in age, BMI, blood counts, CRP, or ESR, confirming appropriate matching. Notably, serum ISM1 in controls showed considerable variability (SD: 698.43 pg/mL), suggesting biological heterogeneity.

**Table 2:** Demographic and laboratory parameters.

Parameter	IGM (*n* = 30)	Control (*n* = 30)	*p*‐Value
Age (years)	38.80 ± 5.49	37.97 ± 5.07	0.778
BMI (kg/m^2^)	24.3 ± 4.5	26.7 ± 3.9	0.632
WBC (×10^3^/µL)	8.76 ± 3.2	8.12 ± 2.8	0.698
Hemoglobin (g/dL)	12.21 ± 1.4	12.61 ± 2.3	0.703
Platelet (×10^3^/µL)	367.9 ± 110.3	343.4 ± 106.9	0.563
ESR (mm/h)	25.58 ± 8.4	22.50 ± 7.6	0.319
CRP (mg/dL)	9.10 ± 3.9	7.82 ± 2.8	0.068

Abbreviations: BMI, body mass index; CRP, C‐reactive protein; ESR, erythrocyte sedimentation rate; IGM, idiopathic granulomatous mastitis; WBC, white blood cell.

### 3.3. ISM1 Levels

**Figure 1:**
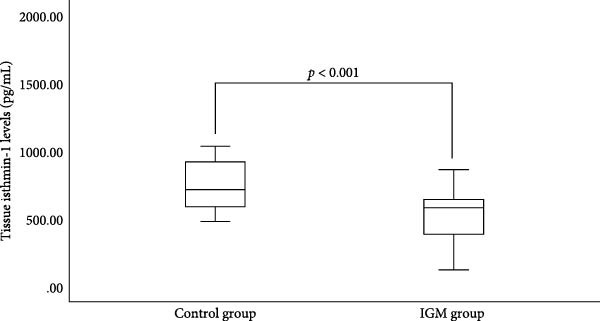
Comparison of tissue isthmin‐1 levels between idiopathic granulomatous mastitis (IGM) patients and control subjects. Tissue isthmin‐1 levels were significantly lower in IGM patients compared to controls.

**Figure 2:**
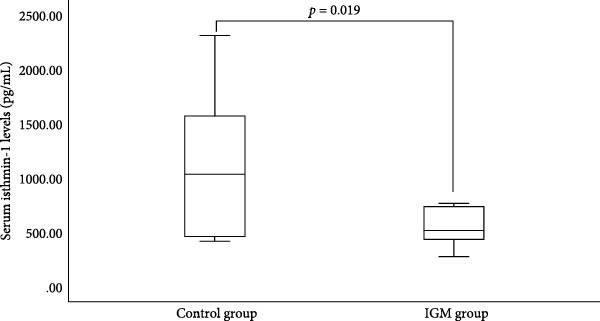
Comparison of serum isthmin‐1 levels between idiopathic granulomatous mastitis (IGM) patients and control subjects. Serum isthmin‐1 levels were significantly reduced in IGM patients compared to controls.

ISM1 concentrations were significantly reduced in IGM patients compared to controls in both tissue and serum samples (Table 3, Figures 1 and 2). Tissue ISM1 was 511.07 ± 188.16 pg/mL in IGM versus 778.24 ± 261.98 pg/mL in controls (*p*  < 0.001). Serum ISM1 was 541.42 ± 191.01 pg/mL in IGM versus 1139.19 ± 698.43 pg/mL in controls (*p* = 0.019).

**Table 3:** Isthmin‐1 levels in study groups.

ISM1 level	IGM (*n* = 30)	Control (*n* = 30)	*p*‐Value
Tissue (pg/mL)	511.07 ± 188.16	778.24 ± 261.98	<0.001
Serum (pg/mL)	541.42 ± 191.01	1139.19 ± 698.43	0.019

Abbreviations: IGM, idiopathic granulomatous mastitis; ISM1, isthmin‐1.

### 3.4. Diagnostic Performance

**Figure 3:**
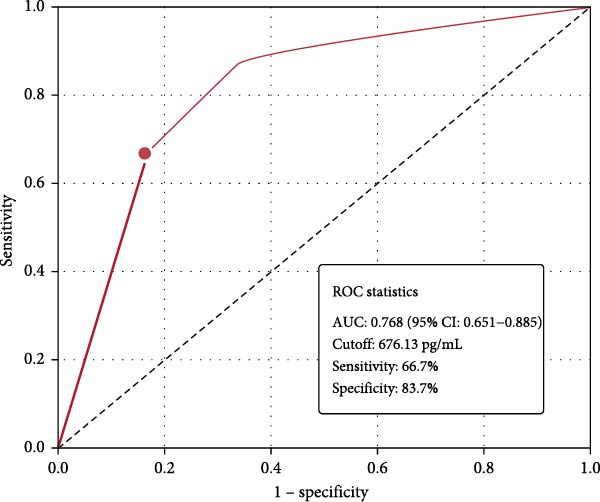
Receiver operating characteristic (ROC) curve for serum ISM1 levels in distinguishing IGM patients from controls. The analysis showed moderate diagnostic accuracy with AUC: 0.768 (95% CI: 0.651–0.885); optimal cutoff: 676.13 pg/mL; sensitivity: 66.7%; specificity: 83.7%. These results indicate that while serum ISM1 shows promise as a potential biomarker, its moderate sensitivity limits its utility as a standalone diagnostic test.

ROC analysis demonstrated moderate diagnostic accuracy of serum ISM1 for distinguishing IGM from controls (Figure 3). The AUC was 0.768 (95% CI: 0.651–0.885, *p* < 0.001). The optimal cutoff value of 676.13 pg/mL yielded 66.7% sensitivity and 83.7% specificity, indicating potential utility as a supportive biomarker rather than a standalone diagnostic test.

## 4. Discussion

This study provides the first evidence of reduced ISM1 levels in both serum and breast tissue of IGM patients, suggesting potential involvement in disease pathogenesis. The significant decrease in ISM1 concentrations indicates a possible link between ISM1 deficiency and the inflammatory processes underlying IGM.

Several mechanisms may explain the observed ISM1 reduction. Experimental evidence indicates ISM1 regulates inflammation through promotion of adiponectin expression and enhancement of macrophage efferocytosis [15, 16]. Impaired clearance of apoptotic cells contributes to granuloma formation and chronic inflammation [17, 18]. Therefore, reduced ISM1 in IGM may impair macrophage function, leading to defective efferocytosis and persistent inflammation.

ISM1 also modulates immune responses through interactions with αvβ5 integrin and GRP78, influencing angiogenesis, apoptosis, and cellular survival pathways [9, 11]. Dysregulation of these pathways could contribute to the abnormal immune response observed in IGM. Additionally, ISM1 affects T‐cell trafficking and immune cell recruitment [12, 19], processes known to be altered in IGM patients [5, 20]. The role of regulatory T cells (Tregs) in resolving inflammation has been well‐documented [21], and alterations in these cell populations may contribute to the chronic inflammatory state observed in IGM.

The moderate diagnostic accuracy of serum ISM1 (AUC: 0.768) suggests potential clinical utility. The high specificity (83.7%) indicates that low ISM1 levels are relatively specific to IGM, though the moderate sensitivity (66.7%) limits its use as a single diagnostic marker. Combining ISM1 with other inflammatory markers or imaging findings could enhance diagnostic accuracy. Recent studies have identified novel inflammation‐associated biomarkers in various chronic inflammatory conditions [22], suggesting that a multi‐biomarker approach may be more effective for IGM diagnosis.

Our findings align with emerging evidence of ISM1’s anti‐inflammatory role in various diseases. Studies have demonstrated reduced ISM1 in inflammatory conditions and its protective effects in lung homeostasis and metabolic disorders [10, 16, 19]. The resolution of chronic inflammation involves complex interactions between macrophages and the extracellular matrix [23], processes that may be disrupted when ISM1 levels are reduced. Recent reviews have highlighted ISM1 as an important adipokine with cardiovascular and anti‐inflammatory properties [24–26], supporting a broader role in immune regulation. The consistent reduction in both serum and tissue samples strengthens the association between ISM1 and IGM pathogenesis.

This study has limitations that warrant consideration. First, the cross‐sectional design precludes the determination of causality between ISM1 reduction and IGM development. Second, using breast reduction patients as controls does not establish whether ISM1 reduction is specific to IGM or represents a general inflammatory response. Third, the modest sample size prevented subgroup analyses based on disease severity or treatment status. Finally, the high variability in control serum ISM1 levels suggests potential confounding factors that require further investigation.

## 5. Conclusions

This case–control study demonstrates significantly reduced ISM1 levels in both serum and tissue of IGM patients, suggesting potential involvement in disease pathogenesis. While serum ISM1 shows moderate diagnostic accuracy with high specificity, its limited sensitivity indicates utility as a supportive rather than a standalone biomarker. Future research should include larger cohorts with other inflammatory breast conditions to establish specificity, longitudinal studies to determine temporal relationships, and mechanistic investigations to clarify ISM1’s role in IGM pathogenesis. These insights could guide the development of novel diagnostic approaches and therapeutic strategies for this challenging condition.

## Ethics Statement

This study protocol was approved by the Firat University Ethics Committee (Approval Number: 26962/24) and conducted in accordance with the Declaration of Helsinki principles, with written informed consent obtained from all participants.

## Conflicts of Interest

The authors declare no conflicts of interest.

## Funding

This project is supported by Fırat University Research Fund Project Number: TF.25.62.

## Data Availability

The data that support the findings of this study are available from the corresponding author upon reasonable request.
